# Effects of individual glucose levels on the neuronal correlates of emotions

**DOI:** 10.3389/fnhum.2013.00212

**Published:** 2013-05-21

**Authors:** Veronika Schöpf, Florian Ph. S. Fischmeister, Christian Windischberger, Florian Gerstl, Michael Wolzt, Karl Æ. Karlsson, Ewald Moser

**Affiliations:** ^1^MR Centre of Excellence, Medical University ViennaVienna, Austria; ^2^Center of Medical Physics and Biomedical Engineering, Medical University ViennaVienna, Austria; ^3^Division of Neuro- and Musculoskeletal Radiology, Department of Radiology, Medical University ViennaVienna, Austria; ^4^Study Group Clinical fMRI, Department of Neurology, Medical University ViennaVienna, Austria; ^5^Department of Clinical Pharmacology, Medical University ViennaVienna, Austria; ^6^Department of Biomedical Engineering, School of Science and Engineering, Reykjavik UniversityReykjavik, Iceland

**Keywords:** functional MRI, hypothalamus, hyperglycemia, hypoglycemia, emotion processing

## Abstract

This study aimed to directly assess the effect of changes in blood glucose levels on the psychological processing of emotionally charged material. We used functional magnetic resonance imaging (fMRI) to evaluate the effect of blood glucose levels on three categories of visually presented emotional stimuli. Seventeen healthy young subjects participated in this study (eight females; nine males; body weight, 69.3 ± 14.9 kg; BMI, 22 ± 2.7; age, 24 ± 3 years), consisting of two functional MRI sessions: (1) after an overnight fast under resting conditions (before glucose administration); (2) after reaching the hyperglycemic state (after glucose administration). During each session, subjects were presented with visual stimuli featuring funny, neutral, and sad content. Single-subject ratings of the stimuli were used to verify the selection of stimuli for each category and were covariates for the fMRI analysis. Analysis of the interaction effect of the two sessions (eu- and hyperglycemia), and the emotional categories accounting for the single-subject glucose differences, revealed a single activation cluster in the hypothalamus. Analysis of the activation profile of the left amygdala corresponded to the three emotional conditions, and this profile was obtained for both sessions regardless of glucose level. Our results indicate that, in a hyperglycemic state, the hypothalamus can no longer respond to emotions. This study offers novel insight for the understanding of disease-related behavior associated with dysregulation of glucose and glucose availability, potentially offering improved diagnostic and novel therapeutic strategies in the future.

## Introduction

The hypothalamus is no longer regarded solely as an organizing center for the integration of somatic and autonomic responses, but as a key organ in the processing of human emotions (Karlsson et al., 2010). Recent studies using event-related functional magnetic resonance imaging (fMRI) have demonstrated that the processing of valence-laden stimuli results in hypothalamic activity patterns comparable to those of the amygdala (Mobbs et al., 2003; Wild et al., 2003; Habel et al., 2007; Watson et al., 2007; Reiss et al., 2008; Schwartz et al., 2008; Derntl et al., 2009), a structure critical for the processing of emotion (Fossati, 2012). Furthermore, the rich reciprocal neural connections between the amygdala and hypothalamus strongly suggest support a role for the hypothalamus in emotion (Herman and Cullinan, 1997; Price, 2003; Hikosaka et al., 2008). For example, the symptoms of the sleep disorder, narcolepsy (Thannickal et al., 2000), strongly indicate hypothalamic involvement in the processing of emotion. Specifically, cataleptic attacks that include a complete loss of muscle tone are the cardinal symptom of narcolepsy and occur predominantly following strong and sudden emotional arousal (Guilleminolt and Fromherz, 2005; Siegel and Boehmer, 2006). This phenomenon is solely contained within the hypothalamus, as cataplexy is fully explained by the loss of hypocretinergic neurons (Siegel, 1999).

Because of its role in sleep and sleep disorders, hypocretin has been the target of numerous research efforts (Van den Pol, 2012). Rodent studies have revealed that hypocretin cells have the highest discharge rates during active wakefulness and exploration and the lowest during REM sleep (Mileykovskiy et al., 2005). Importantly, hypocretin activation seems to be related to positively, as opposed to negatively, valenced arousal states. For example, studies looking at Fos expression show that hypocretin levels are not increased with footshock, a situation of strong negative valence (Furlong et al., 2009). Similarly, hypocretin unit activity decreases in novel situations eliciting withdrawal, but increases with novel situations eliciting exploration (Borgland et al., 2009; Sharf et al., 2010; McGregor et al., 2011). In addition, in humans, low cerebrospinal hypocretin levels are related to depression (Brundin et al., 2009). In an fMRI study, it was shown that there is increased neural activity within the amygdala and the hypothalamus during the processing of both positive and negative stimuli; interestingly, the hypothalamic activation was at the precicse anatomical location of the hypocretin cells (Karlsson et al., 2010). Consistent with a role for hypocretin in the processing of emotion, recent human microdialysis studies have revealed that hypocretin levels are not affected by general arousal; they are elevated with feelings of excitement or laughter, but not with feelings of frustration or sadness (Blouin et al., 2013).

Intriguingly, the *in vitro* activity of hypocretin cells reveals strong inhibition after the administration of physiological levels of glucose (Burdakov et al., 2005, 2006). Over the past few decades, there have been a number of studies suggesting a role for glucose in the modulation of cognitive processes. The beneficial effects of glucose have been observed for a wide range of experimental settings and cognitive tasks across different medical populations and species. In humans, an enhancement effect following glucose administration has been shown for: cognitive performance resulting in a reduction of reaction times (Adan and Serra-Grabulosa, 2010); selective and sustained attention and control (Gagnon et al., 2010; Serra-Grabulosa et al., 2010); continuous performance tests of attention (Flint, 2004); cognitively demanding tasks (Scholey et al., 2001); and learning and memory (for an extensive review see Smith et al., 2011). In clinical populations with severe cognitive deficits, the administration of glucose has been shown to improve cognitive function, for example, memory performance in Alzheimer's disease (Manning et al., 1993; Messier et al., 1997), although there are also negative reports (Craft et al., 1999). Furthermore, in schizophrenia, higher blood glucose levels have been shown to improve verbal memory and declarative learning (Newcomer et al., 1999; Stone and Seidman, 2008).

Based on the evidence of glucose effects on cognitive function, it seems plausible to predict that glucose may also alter mood and arousal, and specifically, emotions. Under stressful conditions, induced by a foot shock, rats exhibit a significant elevation in blood glucose levels (Verago et al., 2001; Farias-Silva et al., 2002; Eguchi et al., 2011) that, according to one study, is comparable to an injection of 100 mg/kg of glucose (Hall and Gold, 1986). In humans, emotionally arousing pictures (Blake et al., 2001) are not only better remembered, but also lead to a higher blood glucose levels compared to neutral pictures, whereas emotional words are better recalled and recognized than neutral words, without a direct link to glucose levels (Ford et al., 2002).

In contrast to the extensive literature about the behavioral effects of glucose, little is known about the neural mechanisms underlying these observations in humans. Most studies investigating the neural correlates of emotions look solely at the role glucose may exert in facilitating memory. Emerging evidence suggests that this cognitive enhancement is mediated by a glucose-induced effect on the hippocampus, since the enhancement is only observed when this cortical structure is critically involved (Parent et al., 2011; Smith et al., 2011). Yet, glucose may also enhance performance by altering amygdala function, as clearly shown by direct glucose administration to the amygdala (Schroeder and Packard, 2003). Studies investigating the role of glucose on memory enhancement using emotional stimuli also found improved memory recall as well as activation differences not only within the hippocampus but also in the amygdala and frontal regions, all related to glucose levels (Brandt et al., 2006, 2010; Parent et al., 2011).

It is, therefore, tempting to speculate that hypothalamic cells are not only responsive to emotional stimuli, but are also both modulated by stimulus valence, as well as glucose levels, and the interaction thereof. In order to address this issue, we used fMRI to investigate the effect of blood glucose levels on the processing of three different categories of visually presented emotional stimuli (funny, neutral, and sad) in seventeen healthy subjects, comparing two different glucose levels. We hypothesized that, while the first level (euglycemia) would yield only modulations induced by emotions, the second level (hyperglycemia) would show the interaction.

## Methods

### Subjects and data acquisition

Seventeen young, healthy volunteers (eight women/nine men; average body weight, 69.3 ± 14.9 kg; BMI, 22 ± 2.7; age, 24 ± 3 years) underwent two fMRI sessions on a 3T TIM Trio scanner (Siemens Medical, Erlangen, Germany), using a 32-channel head coil (25 axial slices; slice thickness 1.9 mm; 128 × 128 matrix; *TR*/*TE* = 2000/40 ms). All experiments were performed at the MR Center of Excellence, Medical University of Vienna, Vienna, Austria, in accordance with the 1975 Helsinki declaration and local ethics regulations.

Subjects were instructed to fast overnight from 8:00 p.m. until scanning the next day, which started between 11:00 a.m. and 2:00 p.m. (no intake of food or beverages, except water). Blood glucose levels were measured before the start of the experiment using an Accu-Check GO (Roche Diagnostics, Vienna, Austria). A venous catheter was used to draw blood for assessing glucose levels during the experiment. Two fMRI sessions were acquired each: (1) in the euglycemic state (before glucose administration); and (2) after reaching the hyperglycemic state (after glucose administration). Each session lasted about 15 min.

### Paradigm

In each of the two sessions, subjects were presented with a set of 30 pictures taken from the complete set comprising 60 pictures. Stimuli featured funny, neutral, and sad content. More specifically, pictures depicted a wide range of scenes, such as car crashes, nature settings, empty office buildings, electrical appliances, humans and animals in comic situations, were presented for 4 s each in randomized order. Two sets were chosen to exclude novelty effects and were presented in randomized order. Stimulus material and stimulus presentation was identical to that of Karlsson et al. (2010). Subjects were asked to passively attend the stimuli without being instructed to fixate on a specific part of the image.

Between the two sessions, a 10% glucose solution (Fresenius Kabi, Graz, Austria) was infused intravenously until a blood glucose level of 160–180 mg/dl was reached. Stimulus presentation order was randomized across subjects. After the fMRI measurements, and when the glucose level had leveled off to a euglycemic state, subjects were asked to rate all presented stimuli using a modified SAM scale comprising the dimensions valence and arousal (Bradley and Lang, 1994). These data were used to verify the selection of the stimuli categories and to exclude systematic differences in individual assessment across the two sessions, using repeated measurement ANOVAs separately per dimension.

### Data analysis

Image preprocessing for all subjects was performed with SPM8 (http://www.fil.ion.ucl.ac.uk/spm/software/spm8/), including slice-timing (Sladky et al., 2011) and motion correction, normalization to an anatomical image template, and spatial smoothing using a Gaussian kernel (FWHM = 8 mm). In addition, realignment parameters were added as nuisance regressors to model for residual motion effects. Statistical analysis was performed at the individual and group levels using SPM8. Single-subject data analysis included calculation of statistical parametric maps using the general linear model with regressors corresponding to the different emotional conditions (funny/neutral/sad).

For second-level analysis, analyses of variances were performed as implemented in SPM8 using the two different fMRI sessions (before/after glucose administration) as one factor, and the emotional categories as the second factor with three levels (funny, neutral, and sad).

In an initial evaluation, the individual glucose level differences between the two sessions were added as a covariate of no interest to obtain neuronal difference effects of sessions and runs, i.e., main effects for emotional categories and sessions.

To account for possible regulation effects due to glucose levels on the session-specific activation patterns and the perception of emotional stimuli, individual session-specific glucose values were included. Using these covariates, a second model was calculated. Both models were thresholded at *p* < 0.001, uncorrected.

## Results

### Glucose levels

Mean glucose levels were 84.35 mg/dl (±7.3 *SD*) for the first session and 177.96 mg/dl (±14.5 *SD*) for the second session. The mean latency for the subjects to reach the predefined glucose levels was 1 h and 16 min (±25 min *SD*).

### Behavioral data

On average, valence measures for the euglycemic sessions were 5.10 (±1.76 *SD*), and 5.22 (±1.85 *SD*) for the hyperglycemic sessions. Arousal levels reached 5.60 (±1.81 *SD*) for the euglycemic sessions, compared to 5.88 (±1.93 *SD*) for the hyperglycemic sessions. There was no significant difference between the measures. Global analysis pooling across the two sessions yielded a significant difference between the three stimulus conditions of funny, neutral, and sad [*F*_(2, 32)_ = 215.075, *p* < 0.000], as well as for arousal [*F*_(2, 32)_ = 40.052, *p* < 0.000]. A detailed analysis, including session analysis, returned a significant result for the factor summarizing the three conditions, but there was no significant effect for session [valence: *F*_(1, 16)_ = 2.232, *p* = 0.155; arousal: *F*_(1, 16)_ = 0.012, *p* = 0.915] nor any interaction effects between session and emotional category [valence: *F*_(2, 32)_ = 2.547, *p* = 0.094; arousal: *F*_(2, 32)_ = 0.940, *p* = 0.401].

### fMRI data

**Figure 1:**
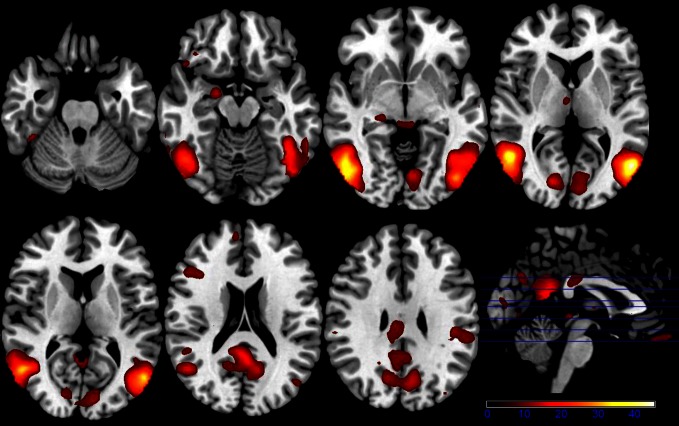
**Axial slices depicting the main effect of emotional categories resulting from a 2 × 3 ANCOVA using the individual glucose level difference as a covariate (*p* < 0.001 uncorrected for whole-brain volume analysis; for a more detailed description of activated brain regions, please see Table 1)**.

**Figure 2:**
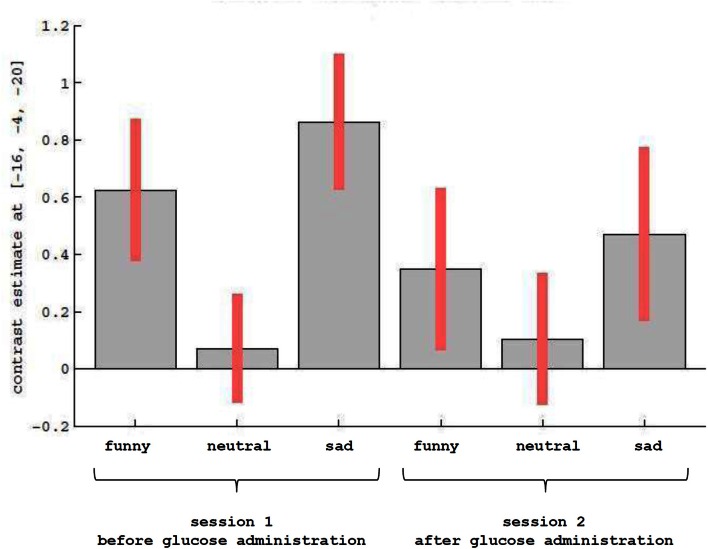
**Description of contrast estimates for the results of the main effect of emotional categories, as shown in Figure 1 in the left amygdala**.

Results of the first 2 × 3 ANCOVA, using the glucose level differences as a covariate, revealed bilateral activation differences in the primary visual cortex and amygdala, depending on the emotional category (see Figure 1 and Table 1 for detailed results). More specifically, the left amygdala activation profile corresponded to the three emotional conditions and was obtained for both sessions, regardless of glucose level. Within this region, positive and negative emotional categories (i.e., funny and sad) showed increased activation compared to baseline, while no alteration from baseline was found for the neutral emotional condition (see Figure 2). A *post-hoc* ROI analysis within the amygdala revealed no significant difference between funny versus sad stimuli across both glucose sessions.

**Table 1:** **Listing for corresponding regions shown in Figures 1, 3, 5, and 6 (*p* < 0.001 uncorrected for whole-brain volume analysis)**.

	**Region**	**Voxels**	***Z*-values**
Figure 1	l. middle temporal gyrus	2969	7.57
	r. middle temporal gyrus	3267	7.29
	l. precuneus	1999	5.16
	r. calcarine gyrus	868	4.88
	r. hippocampus	130	4.36
Figure 3	r. hippocampus	74	4.01
	r. fusiform gyrus	89	3.99
	l. calcarine gyrus	98	3.98
	r. insula	64	3.94
	l. thalamus	49	3.77
Figure 5	l. hypothalamus	3	3.25
Figure 6	r. supramarginal gyrus	45	4.39
	l. cuneus	80	3.94
	r. superior temporal gyrus	37	3.93
	r. caudate nucleus	70	3.84
	r. anterior cingulate gyrus	35	3.75

*Reported are the largest five significantly activated clusters. Clusters were automatically labeled using the AAL toolbox (Eickhoff et al., 2005)*.

**Figure 3:**
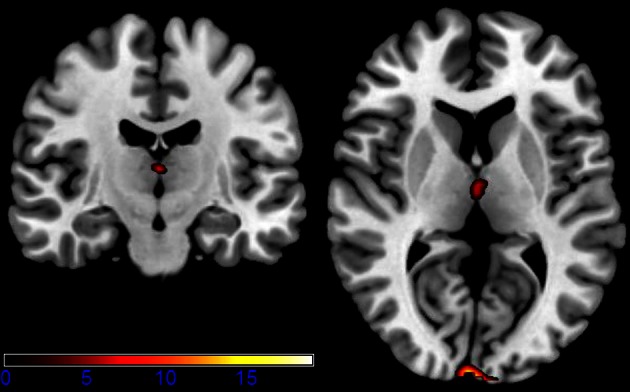
**Axial and coronal slices showing significant activation for the main effect runs (for a detailed description of the analysis, please see text; *p* < 0.001 uncorrected for whole-brain volume analysis; for a more detailed description of activated brain regions, please see Table 1)**.

**Figure 4:**
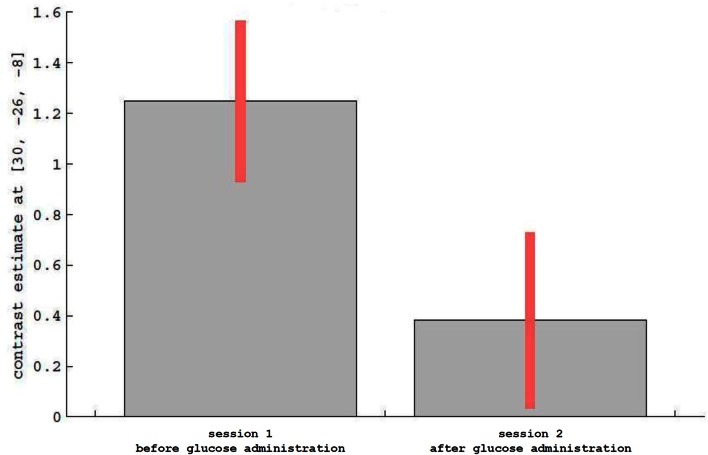
**Description of contrast estimates for the results of the main effect runs in the hippocampus, as shown in Figure 3**.

For the main effect sessions, i.e., contrasting the two sessions regardless of emotional category, higher activation was observed unilaterally in the right temporal cortex, within the hippocampus (see Figure 3 as an activation overview and Figure 4 for contrast estimates in the hippocampus) and the fusiform gyrus, as well as in the thalamus (see Table 1 for detailed results). The first session was the euglycemic state, compared to the hyperglycemic state, the second session.

**Figure 5:**
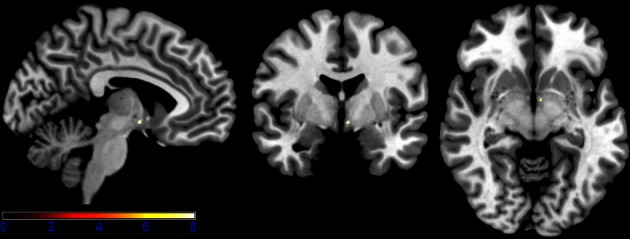
**Shown are sagittal, coronal, and axial slices overlaid with activity clusters corresponding to the interaction effect related to the two sessions and emotional categories (*p* < 0.001 uncorrected for whole-brain volume analysis; for a more detailed description of activated brain regions, please see Table 1)**.

While neutral stimuli did not show any change related to the two sessions, increased activation levels were found for the first session, diminishing in the second session for the emotional categories funny and sad. Finally, summarizing activation changes related to the two sessions and the emotional category differences corresponding to the interaction, effects were found only within the hypothalamus (see Figure 5 and Table 1 for detailed results). A *post-hoc* linear contrast within the interaction showed that the hypothalamus, although differentiating between the emotions in the first session, was not modulated by emotions in the second session.

**Figure 6:**
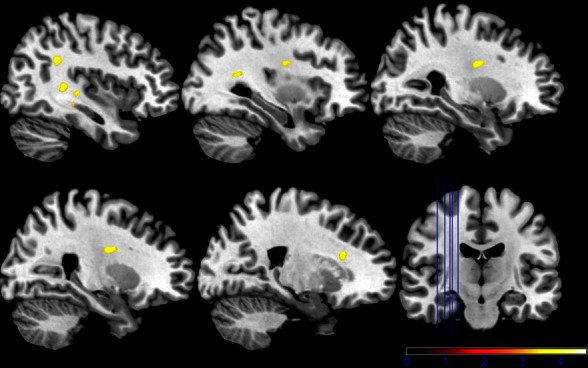
**Shown are sagittal and coronal slices overlaid with activity clusters using individual run-specific glucose levels as covariates (*p* < 0.001 uncorrected for whole-brain volume analysis; for a more detailed description of activated brain regions please see Table 1)**.

The second model using the individual session-specific glucose values as a covariate showed significant activation in the left and right intra-parietal lobules and the right medial cingulate gyrus (see Figure 6) when comparing both sessions with respect to the two glucose levels. Table 1 summarizes all the findings of glucose-related brain activity enhancement in the processing of emotions in young, healthy subjects.

## Discussion

In this study, an analysis of the interaction effect of hypo- and hyperglycemia and stimulus category (funny, neutral, sad) revealed a single activation cluster in the hypothalamus. This finding is in accordance with prior reports on hypothalamic activity during the processing of emotional stimuli (Reiss et al., 2008; Schwartz et al., 2008; Karlsson et al., 2010). Furthermore, as previously reported, the activation cluster correlates precisely with the anatomical location of the hypothalamic hypocretin cells (Karlsson et al., 2010). Karlsson et al. (2010) investigated the effects of funny and sad stimuli compared to neutral stimuli, and demonstrated modulation effects for both the amygdala and hypothalamus. Their observation of a U-shaped activation profile in the hypothalamus concurs with our findings of positive and negative emotional categories revealing high mean beta values, which indicate increased activation in the euglycemic state. However, this modulation vanishes with increased glucose levels. In this hyperglycemic state, the hypothalamus no longer responds to emotions. We, therefore, conclude that at this anatomical site of the hypothalamus, there is a small cluster of cells whose activity is simultaneously modulated by glucose levels and stimulus valence.

fMRI is a non-invasive imaging method that detects transient hemodynamic and functional changes in the brain in response to a variety of stimuli (Bandettini, 2012). The small size of the hypothalamus and its nuclei, combined with low signal changes in fMRI, require specifically optimized protocols (Robinson et al., 2008, 2009) to enable imaging of responses to food-related stimuli in this part of the brain. The use of fMRI to study hypothalamic function in humans has been reported previously (Karlsson et al., 2010). To date, only a few fMRI studies have investigated the involvement of hypothalamic neuronal activity after glucose ingestion in humans (Matsuda et al., 1999; Liu et al., 2000; Smeets et al., 2005, 2007; Vidarsdottir et al., 2007; Purnell et al., 2011).

The hypothalamus is involved in the regulation of food intake and is also responsible for integrating a wide array of hormonal and neural information (Levin et al., 2004), as well as for the evaluation of reward quality and related emotions (Lénárd and Karádi, 2012). This coupling of hypothalamic functioning and glucose ingestion was first shown by Matsuda et al. (1999), who reported on differences in hypothalamic function in lean subjects, *in vivo*, using fMRI to monitor hypothalamic function after oral glucose intake (Matsuda et al., 1999). After glucose ingestion, an increased signal was obtained in the paraventricular and ventromedial nuclei in lean subjects, whereas this inhibitory response was attenuated and delayed in obese subjects. A prolonged dose-dependent decrease in fMRI signal in the hypothalamus after glucose ingestion was confirmed by Smeets et al. (2005), who suggested a possible function for the observed hypothalamic response to changes in blood insulin levels. In addition to fMRI-related findings, other research has revealed increased slow diffusion parameters in the hypothalamus during hypoglycemia induced by fasting (Lizarbe et al., 2013).

The results of Smeets et al. (2005) suggest that the hypothalamus acts as a driving mechanism in areas involved in the processing of emotional stimuli, much like the amygdala, after glucose administration. This finding is also in accordance with experimental reports of hypothalamic functions being altered by glucose intake (Matsuda et al., 1999), and in line with the U-shaped activation curve exhibited within the hypothalamus in response to negative, neutral, and positively valenced stimuli during euglycemic states (Karlsson et al., 2010). Furthermore, this interaction effect of glucose and emotion control was recently recognized in a behavioral study (Niven et al., 2013), which uncovered a correlation of blood glucose levels with poor emotional regulation; the authors hypothesized that glucose provides a limited energy resource upon which self-control relies. The combined demonstration of behavioral data and functional imaging, as realized in our study, enables the investigation of emotional modulation effects related to glucose intake.

A limiting factor in our study is that there was no randomization of glucose level sessions. As euglycemia as a second session would involve the administrations of large doses of insulin to reach a euglycemic state after the hyperglycemic state, we abstained from randomization for safety reasons and unpredictable side effects.

Our findings can pave the way for a more detailed understanding of diseases associated with dysregulation of glucose and glucose availability in the brain, including early metabolic changes starting in childhood (McCrimmon et al., 2012; Reagan, 2012). It has also been shown that glycemic variability significantly impacts mood and quality of life in diabetes (Penckofer et al., 2012), and emotional disorders can negatively affect the course of diabetes (Dziemidok et al., 2011). Moreover, obesity, suggested recently to be a brain-related dysfunction in which reward-driven impulses for food take over response selection systems, was associated with elevations in emotionally driven impulsivity and cognitive inflexibility (Strüber et al., 2008; Delgado-Rico et al., 2012), as well as with emotions that trigger overeating and night-eating (Birketvedt et al., 2002; Koenders and Van Strien, 2011). Those findings might be strongly related to the neural correlates of the processing of emotions. It is reasonable to suggest that the cluster of cells revealed in the current experiment, possibly comprising hypocretin cells, predominantly mediate those effects. Our results offer novel insights into the understanding of disease-related behavior, which could potentially offer improved diagnostic and novel therapeutic strategies in the future.

### Conflict of interest statement

The authors declare that the research was conducted in the absence of any commercial or financial relationships that could be construed as a potential conflict of interest.
